# Cost and cost-effectiveness of a school-based education program to reduce salt intake in children and their families in China

**DOI:** 10.1371/journal.pone.0183033

**Published:** 2017-09-13

**Authors:** Xian Li, Stephen Jan, Lijing L. Yan, Alison Hayes, Yunbo Chu, Haijun Wang, Xiangxian Feng, Wenyi Niu, Feng J. He, Jun Ma, Yanbo Han, Graham A. MacGregor, Yangfeng Wu

**Affiliations:** 1 The George Institute for Global Health at Peking University Health Science Center, Beijing, China; 2 The George Institute for Global Health, Sydney, Australia; 3 School of Public Health, University of Sydney, Sydney, Australia; 4 Institute of Child and Adolescent Health, Peking University Health Science Center, Beijing, China; 5 Changzhi Medical College, Changzhi, Shanxi, China; 6 Wolfson Institute of Preventive Medicine, Barts and The London School of Medicine & Dentistry, Queen Mary University of London, London, United Kingdom; 7 Department of Epidemiology and Biostatistics, Peking University School of Public Health, Beijing, China; 8 Peking University Clinical Research Institute, Beijing, China; Glasgow Caledonian University, UNITED KINGDOM

## Abstract

**Objective:**

The School-based Education Program to Reduce Salt Intake in Children and Their Families study was a cluster randomized control trial among grade five students in 28 primary schools and their families in Changzhi, China. It achieved a significant effect in lowering systolic blood pressure (SBP) in all family adults by 2.3 mmHg and in elderlies (aged > = 60 years) by 9.5 mmHg. The aim of this study was to assess the cost-effectiveness of this salt reduction program.

**Methods:**

Costs of the intervention were assessed using an ingredients approach to identify resource use. A trial-based incremental cost-effectiveness ratio (ICER) was estimated based on the observed effectiveness in lowering SBP. A Markov model was used to estimate the long-term cost-effectiveness of the intervention, and then based on population data, extrapolated to a scenario where the program is scaled up nationwide. Findings were presented in terms of an incremental cost per quality-adjusted life year (QALY). The perspective was that of the health sector.

**Results:**

The intervention cost Int$19.04 per family and yielded an ICER of Int$2.74 (90% CI: 1.17–12.30) per mmHg reduction of SBP in all participants (combining children and adult participants together) compared with control group. If scaled up nationwide for 10 years and assumed deterioration in treatment effect of 50% over this period, it would reach 165 million families and estimated to avert 42,720 acute myocardial infarction deaths and 107,512 stroke deaths in China. This would represent a gain of 635,816 QALYs over 10-year time frame, translating into Int$1,358 per QALY gained.

**Conclusion:**

Based on WHO-CHOICE criteria, our analysis demonstrated that the proposed salt reduction strategy is highly cost-effective, and if scaled up nationwide, the benefits could be substantial.

**Trial registration:**

ClinicalTrials.gov NCT01821144

## Introduction

Cardiovascular disease (CVD), is the leading cause of death and disability worldwide, with approximately 80% of this burden in low-income and middle-income countries and accounting for 42% of total deaths in 2013 in China [1].

Elevated blood pressure (BP) is a major cause of cardiovascular disease and dietary salt intake is the major factor that increases BP [2–4]. The World Health Organization (WHO) has recommended salt reduction as one of the top three priority actions to tackle the global crisis in non-communicable disease [5]. In China, the mean daily salt intake level is more than twice the WHO recommended salt intake (<5.0g/day) [6–9]. China National NCD Action Plan hence in 2012 first time set a goal for population salt reduction [10]. However, reducing salt consumption at a national level is a particular challenge for countries like China where sodium intake is mainly derived from salt added during the preparation of food at home [8]. The School-based Education Program to Reduce Salt Intake in Children and Their Families (School-EduSalt) study provided an innovative approach to reduce salt intake and therefore reduce BP among general population. Its protocol and intervention effect have been published previously [9,11]. The study achieved a significant net reduction in salt intake of 1.9 g/d (27%) in children and of 2.9 g/d (25%) in adults, measured by 24-h urine sodium, over a period of one school semester [9]. This reduction in salt intake was accompanied by a significant net fall in systolic blood pressure (SBP) in adults (2.3 mmHg, 95% confidence interval (CI): 0.04–4.5) [9]. In this paper we report on a cost-effectiveness analysis of the School-EduSalt intervention program, both a trial-based analysis as well as modelled over 10 years scaled up nationwide.

## Methods

### The intervention and trial

The School-EduSalt study was a cluster randomized control trial conducted in 28 primary schools in Changzhi, northern of China in 2013. The schools were randomly assigned (1:1) to either the intervention or the control group after baseline assessments. We selected one grade five class from each school to participate in the study. In total, 592 school students from 14 schools received the intervention program, and 630 students from the other 14 different schools received no intervention. All children in the intervention group were educated on how to reduce salt intake through an 8-lesson salt reduction education program. The lessons were all delivered during one school semester by health education teachers, through the mandatory health education lessons (a 40-minute lesson, every two weeks). Children were assigned homework to further educate their families with tools such as newsletters, calendars, stickers, etc. bearing key educational messages for home cooking as “health ambassadors”. In addition, to monitor the salt consumption in the intervention group, a randomly selected sample of children brought to the classroom their family salt containers (provided centrally) every two weeks, and the teachers measured the weight and calculated with a computer program, and then communicated back the results to families. The details of the intervention were published previously [9,11] with its activities, contents, and materials. To assess the intervention effect, 10 children from each school, along with two adults from each child’s family, were randomly selected to take part in the pre- and post-intervention assessment. In total, 279 children and 553 adults participated in the evaluation assessments. The primary outcome for trial assessment was the change of salt intake as measured by 24-h urine sodium from baseline to the end of the trial and secondary outcome was the change in SBP. As a part of the School-EduSalt trial, the economic evaluation was approved by The Queen Mary Research Ethics Committee (QMREC2012/81) and Peking University IRB (IRB00001052-12072).

### 1.1 Trial-based analysis

As the main results of health outcomes have been reported previously [9], we focused on costing of the intervention and incremental cost-effectiveness ratio (ICER) in this paper. A health sector perspective was adopted and the time horizon for the trial-based analysis was the one semester (3.5 months) duration of the trial. Standard economic costing methods were used to estimate the cost of implementing the School-EduSalt program over the study period [12,13].

The cost of delivering the intervention included personnel costs, transportation and accommodation costs of trainers and trainees, and the cost of educational materials. Personnel unit costs acquired from interviews with the employers and the personnel time from interviews with the employees. The other items were extracted from the financial statements of the project and partner organizations. Costs for research purposes were excluded. The use of antihypertensive drugs for each participant was collected in survey questionnaire and unit prices of these drugs were acquired from local online pharmacies. Salt consumption was estimated by 24-h urine sodium measurements in the trial and costed at prevailing market rates acquired from local supermarkets.

All costs were converted into international dollars (Int$) according to the purchasing power parity conversion factors published by The World Bank (Int$1.00 = 3.52 Chinese yuan) [14].

The intervention cost per participant and per family was determined from the total intervention cost divided by the total number of participants and total number of families in the trial. Incremental cost and incremental effectiveness in the intervention group compared with the control group were determined based on all participants (combining adults and children together), and adults only, separately. ICERs were then calculated from the difference in mean costs divided by the difference on SBP in per participant and per adult. Joint uncertainty of incremental costs and incremental outcomes was estimated by bootstrapping pairs of costs and health outcomes with 1,000 replications [15] and graphically presented on a cost-effectiveness plane. The resulting 1000 pairs of difference in costs and outcomes were used to produce the upper and lower 90% bounds of the ICERs. Based on these bootstrap ICERs, we then estimated the probability that the intervention was deemed cost-effective given a threshold for each additional unit of health outcomes achieved, and presented as a function of a varying threshold in cost-effectiveness acceptability curves [16]. Bootstrapping was done by R software, other analyses were performed using Microsoft EXCEL and SAS 9.3 package (SAS Institute Inc., Cary, NC, USA).

### 1.2 Long-term modelling of population cost-effectiveness

To provide insight into the potential long-term public health impact and benefits of this School-EduSalt intervention at a national level, we assumed a full roll out of this intervention program across China as a long-term government-lead nationwide policy strategy. We adopted the health sector’s perspective and the time frame for base case was 10 years. We simulated the total impact of implementing this program over 10 years from 2016 compared with no program. Findings were presented in terms of an incremental cost per quality-adjusted life year (QALY).

Our efficacy analysis by age group based on the trial data showed that the effect of the intervention in SBP differed by younger (<60 years old) and elder adults (> = 60 years old), a reduction of 0.7 mmHg (95%CI: -1.6 to 2.9) and 9.5 mmHg (95%CI: 2.2 to 16.7) separately. Given that effect on BP lowering was observed mainly in the elderly in the trial and the plausibility of reductions in CVD events was stronger in the elderly, we adopted the conservative modelling strategy of including only the 10 year health effect in the elderly group, but including the full costs of the intervention on all family members.

#### Model overview

**Fig 1:**
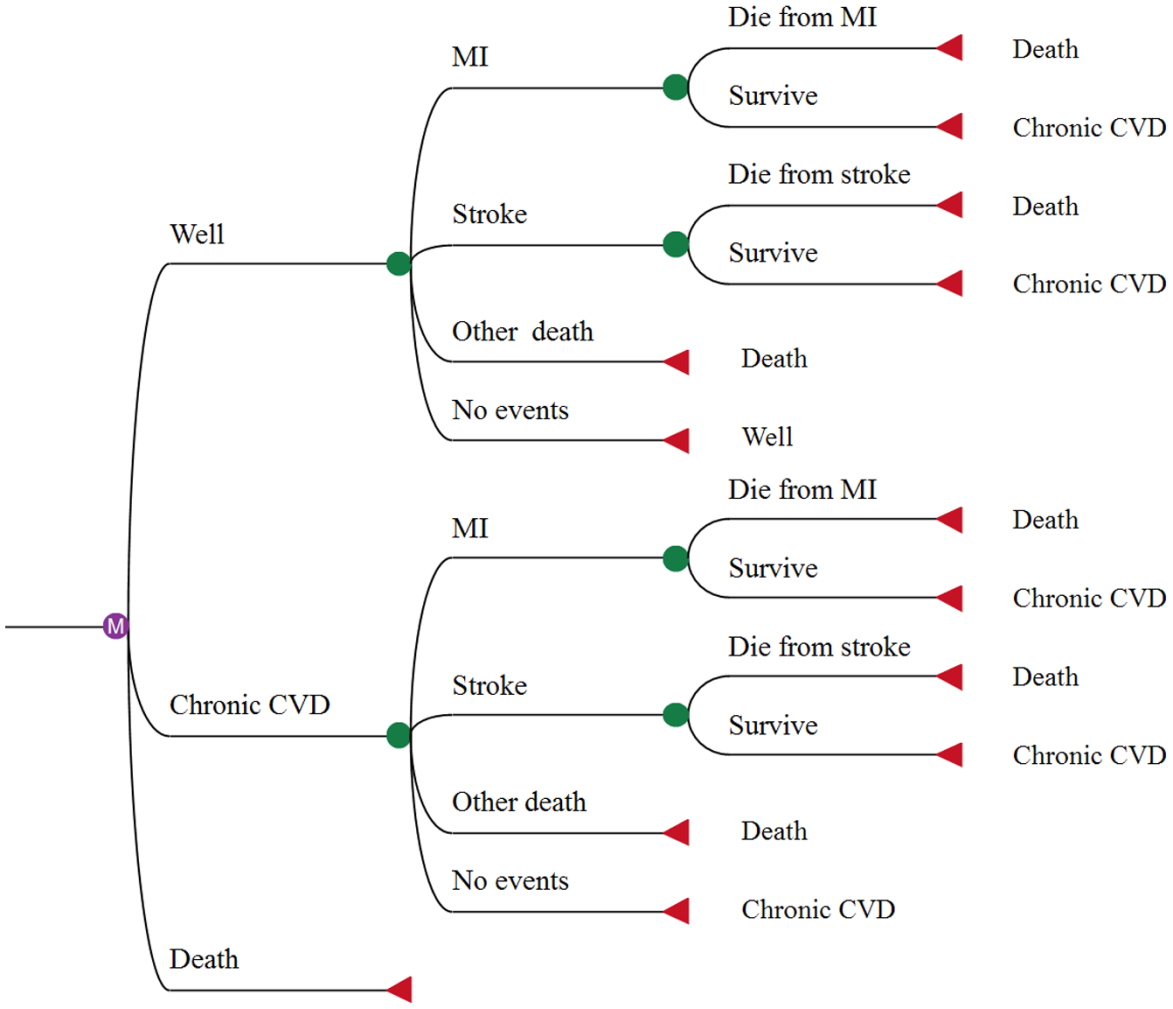
Decision tree structure of the Markov model for each arm (usual care, School-EduSalt program).

We built a 5-state Markov model of incidence, mortality, and costs associated with acute myocardial infarction (MI) and stroke (including ischemic and hemorrhagic stroke) to simulate the cardiovascular outcomes of 165 million families with 10-year-old children across China over a 10-year time frame, derived from the number of in-school students in primary schools [17]. The five health states are: ‘well’, ‘MI’, ‘stroke’, ‘chronic CVD’ and ‘dead’. The “well” state represents people without prior MI or stroke; whereas the “chronic CVD” state represents people with prior MI or stroke. For the initial population, we assigned 4% to start in “chronic CVD” state, consistent with the prevalence of CVD in China among 60–69 years olds (3.96%) [18], the remaining 96% start from the “well” state. MI and stroke are acute states. In each cycle, persons in the well and chronic CVD states may remain in their current health state, or transition to one of other three health states: acute MI, acute stroke, or dead. The decision tree structure of the Markov model for each treatment arm is shown in Fig 1. We used the model to estimate QALYs and 10-year medical expenditures among those elder family members only. Utility and cost weights associated with each state are used to calculate QALYs and costs in each cycle. We used annual cycles for the simulation, and a half-cycle correction was used for annual medical costs and utilities of well state and chronic CVD state. Correction for intervention cost and for the acute inpatient costs and utilities of MI or stroke state was unnecessary as their duration was less than that of a full cycle. We used a discount rate of 3% [19] for costs and effects. The hypothesis was that a reduction in salt intake through its effect on BP would reduce CVD events and deaths, so as to gain QALYs and reduce CVD-related cost. We created the model and performed analyses with TreeAge pro 2015 (TreeAge Software Inc., Williamstown, Massachusetts).

#### Model inputs

The model input parameters, including transition probabilities, effectiveness parameters, cost and utility weights, are shown in Tables 1, 2 and 3 (details see S1 Appendix). These were either drawn from the School-EduSalt trial or derived from the published literatures [17–30] or based on conservative estimates which included: 1) Using only the 10 year total health effect in the elderly group, but including the full costs of the intervention on all family members. 2) Assuming a 50% decrease of the adherence observed in the School-EduSalt trial throughout 10 years, and thereby reduced effectiveness in SBP by 5 mmHg, rather than 9.5 mmHg observed among the elderly in the trial.

**Table 1:** The values of input parameters for Markov models.

Input Parameters	Values (uncertainty*)	Source
***Basic parameters***		
Simulation population	71.2 million elderly from 165 million families in base case	Based on the numbers of in-school students in primary schools in Chinese Ministry of Education website [17]
Starting age	65 years old	Mean age of elderly in School- EduSalt sample.
Proportion of people start from “well”	96% (90%-96%)	[18]
Discount rate	3% (0%-8%)	[19]
***Costs parameters***		
Intervention cost **per family** in the first year	Int$19.04 (±20%)	Table 4.
Ongoing intervention cost in 2–9th year	0	
One hospital admission for stroke	Int$2834.38 (±20%)	China Health and Family Planning Yearbook 2015 [20]
One hospital admission for AMI	Int$7018.75 (±20%)	China Health and Family Planning Yearbook 2015 [20]
MI inpatient days	11 (7–15)	[21]
Stroke inpatient days	28 (23–32)	[22]
Annual medical costs of chronic CVD state	Int$1381.25 (±20%)	Weighted average of rural and urban costs [23] and inflated to 2014 using the average rate of inflation in China from 2003 to 2014 [24].
Per capita total expenditure cost on health in China	Int$734	China Health and Family Planning Yearbook 2015 [20]
***Utility parameters***		
Acute MI state (for inpatient days)	0.87 (0.68–0.97, 0.9179)	[25] [26 GBD 2015]
Acute stroke state (for inpatient days)	0.64 (0.4–0.85, 0.8607)	[27] [26 GBD 2015]
Chronic CVD state	0.872 (0.71–0.95, 0.9106)	[25] [26 GBD 2015]
Well state	1	[27]
Dead state	0	

*data in the () are subjective and used for the sensitivity analyses. Simulation population was derived from the number of grade five students in primary school times the average family size, times the proportion of elderly in those families, and then times 10 years of time. Int$1.00 = 3.52 Chinese yuan. GBD: Global Burden of Disease study.

**Table 2:** The values of input probability parameters for Markov models.

*Transition Probabilities in Well State*	*Values*	*Source*
First-ever incidence of stroke per annum		
in 60–69 years in 70–79 years	1440 /100,000 2280 /100,000	[20, 28]
First-ever incidence of AMI per annum		
in 60–69 years in 70–79 years	325 /100,000 490 /100,000	[20, 29]
28-day mortality risk of first-ever stroke		
in 60–69 years in 70–79 years	20% 20%	[30,31]
28-day mortality risk of first-ever AMI		
in 60–69 years in 70–79 years	42% 45%	[29]
Non-CVD mortality per annum in well state		
in 60–69 years in 70–79 years	1193.5 /100,000 1905.4 /100,000	[1, 20]
***Transition Probabilities in Chronic CVD State***		
Recurrent incidence of stroke per annum		
in 60–69 years in 70–79 years	5760 /100,000 9120 /100,000	Assumed 4 (3~5) times of that in well state.
Recurrent incidence of AMI per annum		
in 60–69 years in 70–79 years	1300 /100,000 1960 /100,000	Assumed 4 (3~5) times of that in well state.
28-day case-fatality of recurrent stroke		
in 60–69 years in 70–79 years	28% 28%	Assumed 1.4 (1~2) times of that in well state [31]
28-day case-fatality of recurrent AMI		
in 60–69 years in 70–79 years	58.8% 63%	Assumed 1.4 (1~2) times of that in well state [31]
Non-CVD mortality per annum		
in 60–69 years in 70–79 years	1432.2 /100,000 2286.5 /100,000	Assumed 1.2 (1~1.5) times of that in well state

**Table 3:** Assumed effect size on SBP by three scenarios and the corresponding annual RRs for Markov model.

Scenarios	Simulation years	Assumed SBP reduction	RRs for 10 mmHg lower SBP from APCSC study			Annual RRs for Markov models corresponding to assumed SBP reduction	
			Age group (years)	RRs in AMI (95% CI) [32, 33]	RRs in stroke (95% CI) [32]	RRs in AMI (uncertainty*)	RRs in stroke (uncertainty*)
Worst case	at 1^st^ year 2^nd^-10^th^ year	5 mmHg 0 mmHg				0.9757 1.0000	0.9670 1.0000
Base case	1^st^-5^th^ year 6^th^-10^th^ year	5 mmHg 5 mmHg	60–69 ys 70–79 ys	0.709 (0.667–0.761) 0.753 (0.709–0.801)	0.626 (0.606–0.646) 0.715 (0.685–0.745)	0.9757 (0.9715–0.9807) 0.9800 (0.9757–0.9843)	0.9670 (0.9649–0.9693) 0.9764 (0.9733–0.9792)
Best case	1^st^-5^th^ year 6^th^-10^th^ year	9.5 mmHg 9.5 mmHg				0.9544 0.9623	0.9383 0.9556

RR: Relative Risk; SBP: Systolic Blood Pressure; CI: Confidence Interval.

Base case: Assuming a 50% decrease of the adherence observed in the trial and thereby reduced effectiveness in SBP by 5 mmHg. Annual RRs were derived from the RRs for 10 mmHg lower SBP in this table according to the assumed SBP reduction and were annualized. Data in the () are used for the sensitivity analyses, which are calculated from the 95%CI of RRs. Aged 65–69 years at 1^st^–5^th^ year of the simulation and 70–74 years at 6^th^–10^th^ year of simulation.

#### Sensitivity analysis

We conducted multiple one-way sensitivity analyses. The first is for the sustainability of effectiveness on SBP. Two scenarios were assumed: the worst scenario where the 50% of intervention effect diminishes immediately after intervention ended versus the best scenario where the intervention effect persists over the 10 years. The assumptions of effectiveness on SBP for each scenario and the corresponding relative risks (RRs) on AMI and stroke are given in Table 3. We then assessed the impact on the ICER and QALYs of using the utilities from Global Burden of Disease 2015 study [26]. We also varied the time horizon to 15 and 20 years to understand how the impact of the intervention will vary by time horizon.

In addition, we performed multiple one-way sensitivity analyses to assess the impact of uncertainty of other key variables on base case modelling results by varying these model parameters. The ranges of these parameters are shown in Tables 1 and 3 in brackets to show their uncertainty and the impact on the ICER and QALYs of varying these key parameters is displayed as Tornado diagrams.

## Results

### 1.1 Intervention cost

The costs of the intervention were itemized in Table 4. Overall, the total cost of implementing the School-EduSalt intervention program in 14 classes was approximately Int$1,358, and the average cost was Int$19.04 per family (Table 4) and Int$6.35 per participant (Table 5).

**Table 4:** Costs of School-EduSalt intervention including set-up costs and running the program for 1 semester.

Categories	Notes	Rate (Int$)	Quantity	Costs (Int$)
***Intervention costs on 592 families***				
**Personnel costs by roles**			hours	
Health education teachers	(1)Receive training from professional researchers as trainees;	8.07	96	774.43
	(2)Deliver education courses to all students in intervention classes.	8.07	210.6	1699.15
Trainers for education teachers	Professional researchers who trained health education teachers.	9.94	72	715.91
Principles of primary schools	Coordinating the implementation of program in their schools.	7.39	49	361.93
Class teachers-in-charge	Time spent on coordination, organization of activities in education courses, parents meeting, etc.	4.94	151.2	747.44
***subtotal***				**4298.86**
**Materials**			numbers	
Educational books	One for each family.	1.70	592	1009.09
Family education newsletters	Four issues for each family.	0.34	2368	807.39
Posters hang in home	One for each family.	0.82	592	487.78
Posters hang in classroom	Three different posters for each classroom.	6.53	42	274.43
Fridge magnets	One for each family.	0.34	592	201.70
Teaching aids	One laser pens for each health education teacher and several recorder pens, etc.			186.36
Logistics	For shipping all above materials from project center to study site.			97.16
***subtotal***				**3063.92**
**Travel expenses**	Transportation and accommodation fees for the trainers.			**2354.83**
***Costs for monitoring cooking salt use (estimated from 141 randomly selected families)***				
**Personnel costs by roles**			hours	
Class teachers-in-charge	Time spent on biweekly monitoring salt consumption	4.94	44.8	**221.59**
**Materials**			numbers	
Salt utensils	One salt container and one 2g salt-control spoon for each family.	4.12	141	580.97
Logs of high salt food	One for each family.	3.92	141	552.84
Scales	One for each class to weigh salt consumed.	9.09	14	127.27
Stickers	One large size stickers for each family.	0.51	141	72.16
***subtotal***				**1333.24**
**Total intervention cost**				**11272.44**
**Total intervention cost per family**				**19.04**

1 Int$ = 3.52 Yuan.

**Table 5:** Trial-based cost effectiveness results over 3.5 months duration of the trial.

Population Groups	Intervention cost (Int$) per participant (C1)	Individual cost on average (Int$)		Incremental cost (Int$) compare to control (C4)	Effectiveness	ICER (90% CI)
		on anti-HTN drug* (C2)	on salt (C3)		on SBP, mmHg (C5)	(Int$/mmHg) (C6)
**All**						
Intervention	6.35	2.16	0.93	4.93	1.8^9^	2.74 (1.17–12.30)
Control	0	3.24	1.27			
**Adults only**						
Intervention	9.52	3.26	0.93	7.54	2.3^9^	3.28 (1.35–14.04)
Control	0	4.90	1.27			
**Elder adults only**						
Intervention	44.13	13.21	0.93	37.82	9.5	3.98
Control	0	19.19	1.26			

HTN: hypertension; SBP: systolic blood pressure, mmHg; ICER: Incremental Cost-Effective Ratio; CI: confidence interval. 1 Int$ = 3.52 Yuan.

* The cost on anti-HTN drug were calculated on the basis of the dosage, unit price, and duration of intervention. The cost on salt was based on the daily amount of salt intake estimated by 24hr urine sodium, the market price, and duration of intervention. The average costs in the table were further divided by the number of participant in corresponding groups.

C4 = (C1 + C2 + C3) in intervention–(C1 + C2 + C3) in control; C6 = C4÷C5;

### 1.2 Trial-based ICER

**Fig 2:**
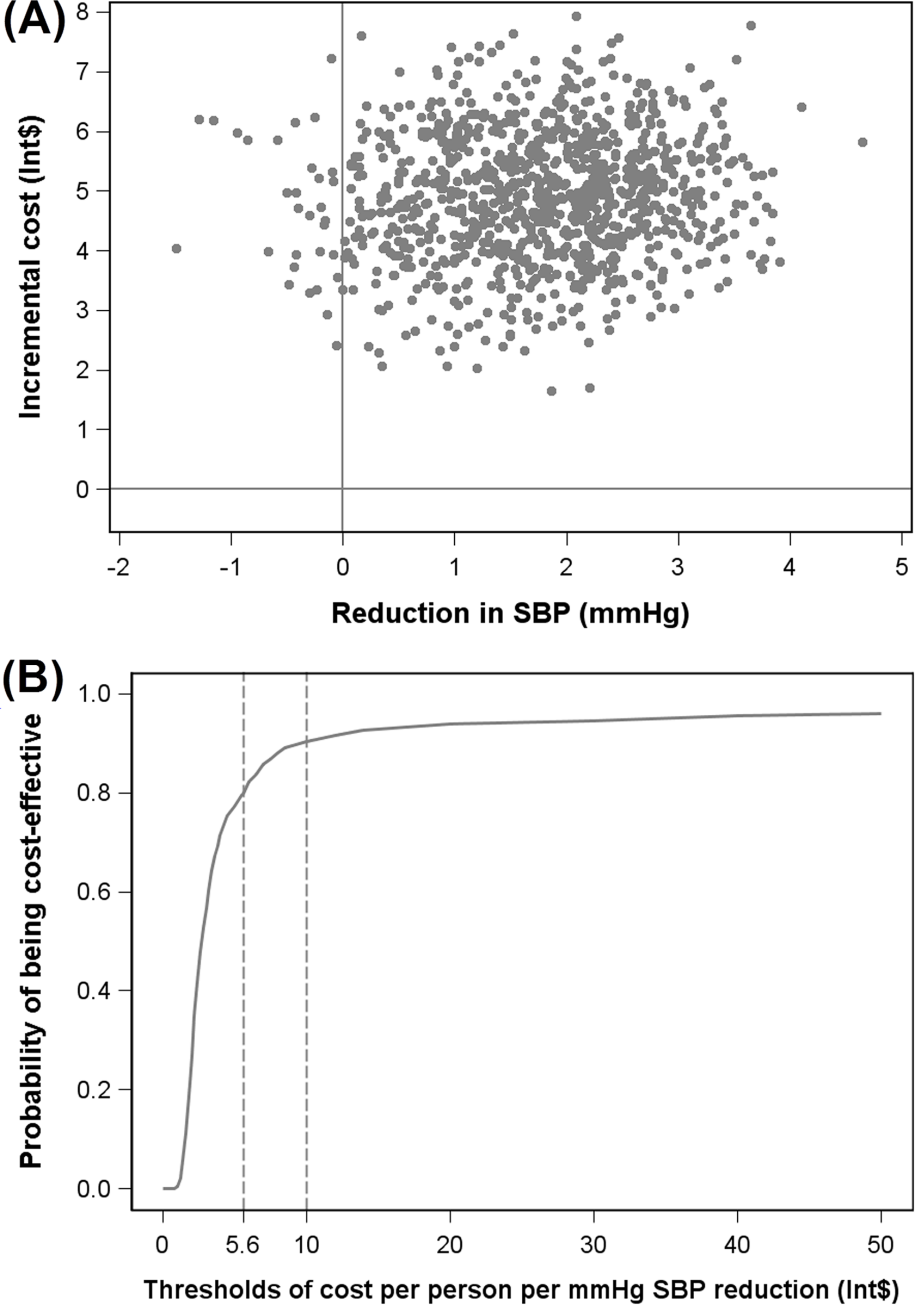
Cost-effectiveness of the program for SBP reduction from bootstrapping. (A) Incremental cost-effectiveness plane shows the joint distribution of 1000 pairs of incremental cost and incremental effect on SBP in School-EduSalt intervention compared with no intervention. (B) Cost-effectiveness acceptability curve for per mmHg SBP reduction.

Trial-based cost-effectiveness analysis were presented in Table 5. The joint bootstrap distribution of the difference in SBP and cost was displayed in a cost-effectiveness plane in Fig 2A, and the relationship between the threshold and the probability of the intervention being cost-effective was shown in a cost-effectiveness acceptability curve in Fig 2B. The acceptability curve demonstrates that the intervention has an 80% probability of being cost-effective relative to the control at a threshold of Int$5.6 per mmHg reduction in SBP.

### 1.3 Model-based analysis

The results from Markov simulation for base case and other scenarios were presented in Table 6, which are all highly cost-effective, even cost-saving in the best scenario. In the base-case scenario, implementing the School-EduSalt intervention program nationwide for 10 years would avert 42,720 AMI deaths and 107,512 stroke deaths in elder adults of China. This represents a gain of 646,496 life-years and 635,816 QALYs. The program would be expected to result in an ICER of Int$1,358 per QALY gained and Int$1,335 per life-year gained.

**Table 6:** The cumulative health and economic consequences implementing the School-EduSalt program compared with no this intervention from Markov models and scenario analysis and beyond 10, 15, and 20-year time horizon.

Elderly reached	Incremental cost, billion (Int$)	QALYs gained	Incremental cost (Int$) per QALY C1/C2	Averted new incidence		Averted deaths of		
				AMI	Stroke	AMI	Stroke	
	C1	C2		C4	C5	C6	C7	
**Base case (assumed 50% of adherence in the trial resulted in halved the effectiveness in SBP in elderly to 5 mmHg and beyond 10-year time horizon)**								
71.2 million	0.86	635,816	1,358	81,880	459,240	42,720	107,512	Highly cost-effective
**Sensitivity analysis on different scenarios of effectiveness in SBP beyond 10-year time horizon**								
**Best scenario (9.5 mmHg effectiveness on SBP in elderly lasted over 10 years)**								
71.2 million	-1.14	1,190,749	-954	154,504	859,384	79,744	200,784	Cost-saving
**Worst scenario (5 mmHg effectiveness on SBP in elderly in year 1 (including 3.5 months intervention period), then drop to 0 in year 2–9)**								
71.2 million	2.50	230,546	10,837	16,376	91,848	7,832	21,360	Highly cost-effective
**Sensitivity analysis for different time horizon**								
**Beyond 15-year time horizon**								
106.8 million	0.29	2,182,992	132	196,512	1,105,380	119,616	371,664	Highly cost-effective
**Beyond 20-year time horizon**								
142.4 million	0.69	4,378,942	158	304,736	1,730,160	193,664	626,560	Highly cost-effective
**Sensitivity analysis for varying utilities for health states from GBD data**								
**Use utilities from GBD 2015 data**								
71.2 million	0.86	600,002	1,439	81,880	459,240	42,720	107,512	Highly cost-effective

QALYs: quality-adjusted life years; AMI: acute myocardial infarction; GBD: Global Burden of Disease study. 1 Int$ = 3.52 Yuan.

**Fig 3:**
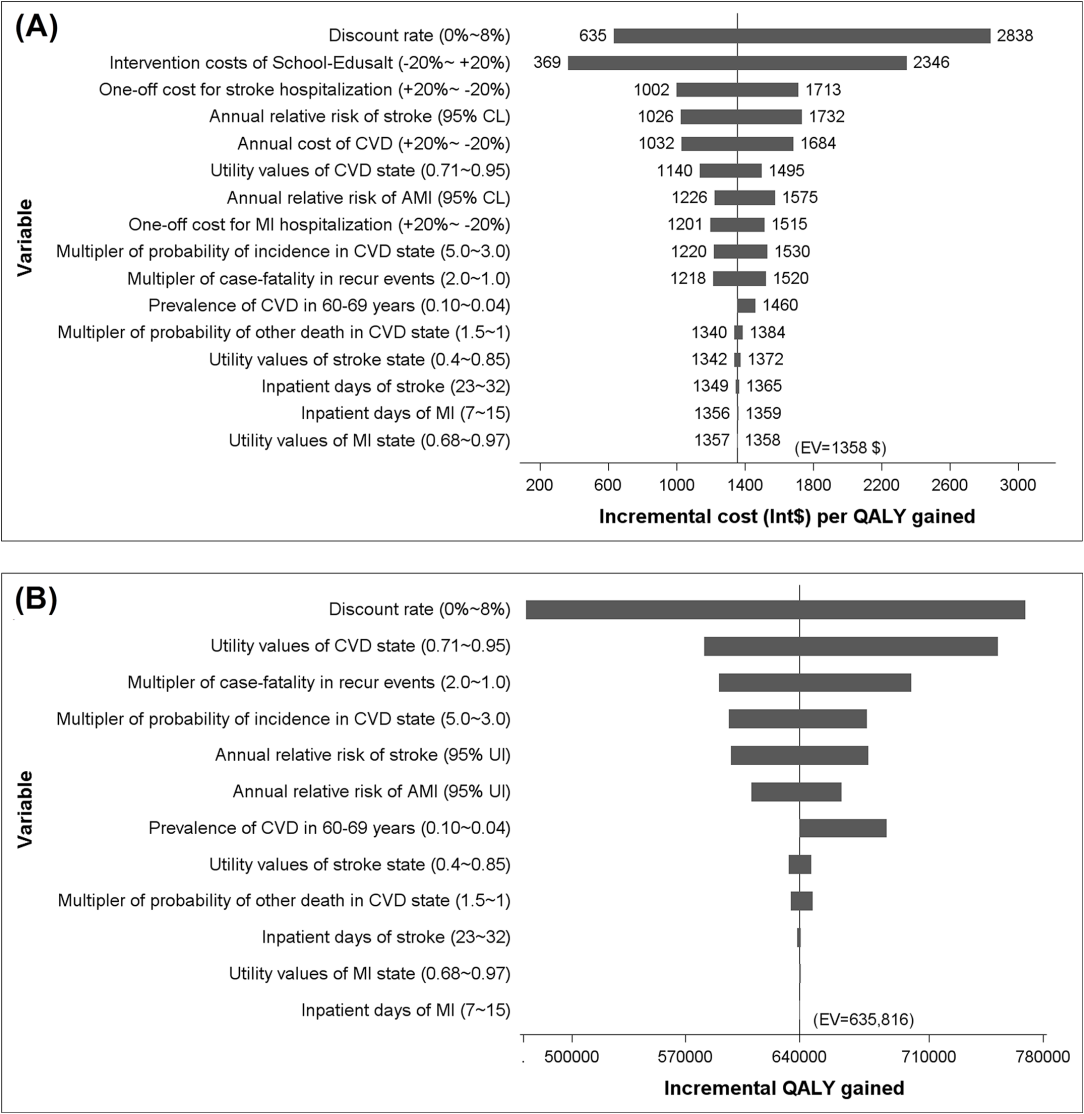
Tornado diagram, showing the influence on outcomes in the base case associated with uncertainty in one variable while all others are stable. The vertical axis denotes the base case. The bars present the changes from the base case. The dollars labelled beside the bars are the corresponding outcome values. Panel (A) denotes the base case incremental costs per QALY gained; Panel (B) denotes the base case incremental QALYs.

The Tornado diagrams in Fig 3A and 3B illustrated the impact of varying other key input parameters on the incremental costs per QALY gained and on the QALYs gained. It shows that the base case finding that the intervention is highly cost-effective is robust against variations in key assumptions.

## Discussion

Whilst there has been substantial evidence of the cost-effectiveness of community based salt reduction strategies [27,34–38], this is the first study to address this question using primary panel data, drawing from a cluster randomized controlled trial.

In the study population, implementing the education program cost Int$2.74 (1.17–12.30) to achieve 1 mmHg net reduction in SBP per participant (adults and children together) and Int$3.28 (1.35–14.04) per adult participant. If the School-EduSalt program were to be rolled out to the whole country and run for 10 years in grade 5 of primary schools from 2016–2025, in the base scenario, about 71.2 million elderly from 165 million households would be directly reached, accounting for 35% of all 60 years old and above in China. The results from the base case Markov model, which assumed adherence to 50% of the reduction in salt intake observed in the trial, indicates that such investment would achieve aggregate health gains of 635,816 QALYs through reduced 541,120 AMI or stroke cases and further 150,232 CVD-related deaths. Given that the gross domestic product (GDP) per capita of China in 2014 (Int$13,253) [39], the proposed intervention can be deemed highly cost-effective (Int$1,358 per QALY gained) when benchmarked against the conventional WHO-CHOICE threshold of an incremental cost per QALY gain of a single the country’s annual GDP per capita [40]. Even with the assumed worst scenario, the intervention would be still highly cost-effective (Int$10,837 per QALY gained) (Table 6). Sensitivity analysis (Fig 3A) indicated that the finding that the proposed intervention is highly cost-effective is robust to variation in key parameters.

To understand the nationwide generalizability of our study, in terms of the coverage, we compared the average size of school classes in the study with that of China, which was 42 vs 40 students per school class in grade 5 [17]. We also compared the mean proportion of 65-year-old and above in Shanxi Province (7.6%) where the trial conducted with the national mean proportion (8.9%) [17]. All these comparisons indicated that our estimates on national scaling up are conservative. To account for the variations of cost by province, we varied all the cost parameters by ±20% in our sensitivity analysis. In addition, the uniformly very high enrollment rate of school-age children in primary schools in China (99.9% in 2015, ranging from 98.9% to 100%) [17] suggests regional differences in roll out will not be significant. Moreover, all primary schools in China are required by the Ministry of Education to provide the health education lessons every 2 weeks in each school term, each for 40 minutes, our proposed intervention does not change the schools’ curriculum and the way of running schools, but is a replacement in first school term of these mandatory lessons. The School-EduSalt intervention was designed to fit in the government regulations and thus sustainable if rolled out.

The findings in this paper highlight the strong economic case for investment in nationwide salt reduction programs is consistent with evidence elsewhere. For instance, the UK, leading the world on salt reduction, has successfully developed a program of voluntary salt reduction in collaboration with the food industry along with consumer awareness campaign. The average salt intake, as measured by 24-h urinary sodium, in people aged 19–64 years has fallen from 9.5 in 2000/01 to 8.1 g/d in 2011 (15% reduction) [41]. Accompanying this was a fall in average SBP of 3.0 mmHg and in CVD mortality by 2011 in population aged≥16 years [41]. NICE (the National Institute for Health and Clinical Excellence) estimated that the UK salt reduction campaigns, which cost £15 million, led to about 9000 fewer CVD deaths per year, saving the UK economy more than £1.5 billion per annum [42]. In Finland, through collaboration with the food industry, mass media campaign, and implementing salt labeling legislation, the salt reduction program resulted in a one-third decrease in the average salt intake from approximately 12 g/d in 1979 to less than 9 g/d in 2002 [43], accompanied by a more than 10 mmHg fall in the population average of both systolic and diastolic blood pressure, and a pronounced decrease of 75% to 80% in both stroke and coronary heart disease mortality [43]. Given these success, a global action group named World Action on Salt and Health (WASH) was established in 2005 to encourage actions on salt reduction worldwide [44], which has been supported to date by 83 countries [45]. Several modeling studies have indicated that a reduction in salt intake through above strategies in US population [34], Norwegian [35], Canadian [36], and worldwide [37,38], is very cost-effective. And Asaria et al suggested a reduction in salt intake is more or at the very least just as cost-effective as tobacco control in reducing CVD for 23 low- and middle-income countries [38]. Moreover, our study showed that our proposed salt reduction strategy is more cost-effective than antihypertensive treatment (Int$ 13,000 per QALY gained) [46].

Recently several publications [47–50] regarding the cost-effectiveness analysis of these strategies added to the existing literatures to provide additional modelling-level evidence that the interventions for reducing salt in the food supply would provide large health gains and also large cost-savings or cost effective for a health system in England [47], India [48], New Zealand [49], and Eastern Mediterranean countries [50]. However, these “supply side” strategies are likely to be less relevant for countries like China where the primary source of dietary salt is home-cooked food [8]. As far as is known, to date, no country has demonstrated the net cost-effectiveness of a successful program where salt intake has fallen due to consumers using less salt during cooking. The current study provides the evidence of the affordability of School-EduSalt program and a template for policy-makers to upscale their efforts to reduce salt consumption across the country. Due to cultural differences, differences in costs of living and family sizes generalizing to western countries should be made with caution.

Moreover, in the model-based analysis, we adopted a conservative strategy. First we simulated the cardiovascular outcomes of only AMI and ischemic and hemorrhagic stroke, regardless of other coronary heart diseases, subarachnoid hemorrhage and transient ischemic attack. Second we considered only the benefits in elderly family members (age over 60 years). Additional benefits to children and young adults were not quantified in this paper but had been demonstrated in previous studies [51,52]. Third, we assumed an erosion of program effectiveness of 50% over 10 years. Fourth, it was estimated that 35% of all stroke survivors would suffer from a permanent physical disability [53], whose vast direct non-medical cost on care givers were not accounted for in the current model. Finally, the nationwide and long-term rolling out of the intervention will generate a much greater change of the social environment for salt reduction than that in the School-EduSalt study according to the social cognitive theory [54]. This is because the trial design necessitated limits to be placed on mass communications to minimize contamination. As such limitations would not be present in a nationwide rollout, it is likely that the size of the effect of BP lowering observed in the School-EduSalt study could be enhanced during roll out.

The study had a few limitations. Firstly, the health outcome of this trial was the difference of SBP between groups, which was intermediate endpoint. As such the trial-based cost effectiveness analysis was not anchored to the hard endpoint such as hospitalization or death. Secondly, we did not have direct evidence as to how long the drop in SBP observed from this 3.5 months trial would remain. As such we used various assumptions about the erosion in treatment effect in terms of the fall on SBP over time—and settled on the conservative assumption of a 50%. Thirdly, we could not account for the eventuality of sudden death as no reliable data exists but it is likely to be low. Lastly, our modeling was based on the trial in one location of China. How China’s vast variation in socio-economy and geography would affect our results is difficult to confidently predict. Usually, an intervention successful in a small or medium city should be much more generalizable than those developed in megacities where resources are much more fluent. Further, China’s successful universal compulsory education provides the necessary platform for universal coverage and compliance given that the School-EduSalt intervention was designed to fit into the existing curriculum.

## Conclusion

This study demonstrated that the School-EduSalt intervention program for salt reduction is of low cost and highly cost-effective. The nationwide implementation program over 10 years is predicted to prevent at least 42,720 AMI deaths and 107,512 stroke deaths and achieve significant medical cost savings.

## Supporting information

S1 Appendix Supporting technique appendix for modeling. (DOCX)
