# Supplementary material for: Cost and cost-effectiveness of a school-based education program to reduce salt intake in children and their families in China
Source: PLoS One. 2017 Sep 13;12(9):e0183033. doi: 10.1371/journal.pone.0183033 (PMC5597122; doi:10.1371/journal.pone.0183033)
Supplement: S1 Appendix — (DOCX) [file pone.0183033.s001.docx]

**Supporting appendix for modeling**

This appendix provides further methodological details for modelling analysis.

**Contents:**

**Section 1. Estimation of population over 60 years for the simulation cohort**

**Section 2. Cost parameters**

**Section 3. Utility parameters**

**Section 4. Effectiveness parameters and relative risks**

**Section 5. Transition probabilities between states**

**Section 6. References**

**Section 1. Estimation of population over 60 years for the simulation cohort**

We modelled a roll out of this education program over 10 years from 2016. To be conservative, we simulated the health outcomes only on older family members. We estimated the total number of elderly in China that could be covered by the intervention during 10 years in the following way. Based on the national total number of grade five students in primary schools [1], it was estimated that there will be about 165 million children aged 10-years in primary schools across China between 2016-2025 who along with their families could receive the intervention. Based on School-EduSalt trial, the average family size was 3.925 and the older adults aged 60 years or above accounted for 11% among family members. As a result, the total number of older adults over 10 years who could be targeted was about 71.2 million (≈165 ×3.925×11%).

Each year about 16.5 million children and their families will get the intervention in the base-case simulation. To be simpler, we assumed that we treat the whole 165 million families over ten years in year 1, and no further intervention and ongoing cost in year 2-9.

**Section 2. Cost parameters**

It was assumed that people who suffered acute MI or stroke events would receive acute treatment and incur medical expenditure regardless of subsequent death. In the base-case analysis, the cost of acute treatment of AMI 24,706 Yuan (Int$7018.75) in 2014 was from China Health and Family Planning Yearbook 2015 [2]. The cost of acute treatment of stroke 9,977 Yuan (Int$2834.38) was a weighted average of the 2014 cost of ischemic and hemorrhagic stroke, accounting for the numbers of discharged patients from China Health and Family Planning Yearbook 2015 [2]. Non-fatal CVD would also incurs annual medical costs. The annual cost per capita of chronic CVD state in 2014 was estimated about 4,862 Yuan (Int$1381.25) which was based on 2003 data [3], accounted for weighted average of rural and urban costs and inflated to 2014 using the average rate of inflation in China from 2003 to 2014 [4].

All Yuan were converted into international dollars (Int$) according to the purchasing power parity conversion factors by The World Bank (Int$1.00 = 3.52 Chinese yuan).

Based on the intervention cost per family of Int$19.04 (67.03 Yuan) as shown in Table 4 in the manuscript, for base-case scenario, we aggregated all the investment spent on whole 165 million families into 71.2 million elderly. It resulted in that the intervention cost Int$44.13 per elder adult (Table 4 in the manuscript).

**Section 3****. Utility parameters**

The health-related quality of life (QOL) weights, we called as utility, for each health state were taken from the literature [5,6] and listed in Table 1 in the manuscript. The utility for acute state of stroke was elicited from Smith-Spangler CM et al [5]. For other health states, we chose those utilities based on the time tradeoff (TTO), a direct QOL assessment method from Tengs TO et al [6]. Such as, the utility for acute state of MI was elicited from line NO. 596 in Appendix A [6], which was drawn from a sample of 2,579 patients and patient proxies based on TTO method. The utility for chronic CVD state (0.872) were averaged by that of the chronic states after MI or after acute stroke which elicited from line NO. 659, 388, 392, 393, and 407, respectively, in Appendix A of Tengs TO et al [6], and weighted by the estimated ratio 1:2 between MI and stroke in China. Acute MI and acute stroke are acute states representing 11 hospital days for AMI and 28 hospital days for stroke. The survivors will transit to chronic CVD state after discharge and apply the utility of chronic CVD state. QALYs were determined from utility multiplied by time in state.

**Section 4. Effectiveness and relative risks (RRs) on CVD**

**RRs attributed to lower SBP**

The RRs for modelling listed in Table 3 in the manuscript were based on a meta-analysis of the Asia Pacific Cohort Studies Collaboration (APCSC) [7], which included 14 of 37 cohort studies from mainland China [7]. Several recent meta-analyses of cohort studies have published the age-specific estimates of RR for SBP on cardiovascular outcomes, such as APCSC [7] and Prospective Studies Collaboration (PSC) [8], with 958,074 participants mainly from Europe and United States. Taking account of the association between BP and stroke in Asia was slightly steeper than in Europe [8], and the Unites States [8] after standardization for age from regional comparisons in APCSC and PSC, we conservatively chose the estimates of RR from APCSC study [7]. Furthermore, when age-specific RRs were available, a clear age gradient was observed [7,8], with smaller RRs in older ages, but there has been no evidence in any of the overviews that the strength of association between BP and stroke varied by sex or for fatal and nonfatal stroke events, we thus adopted the age-specific sex aggregated RRs.

From above, the estimated RR of total stroke for 10 mmHg lower usual SBP in 60-69 years group was 0.626. This represented the weighted geometric mean of RR reduction for hemorrhagic stroke (0.40) and ischemic stroke (0.35) [7,9]. The RR of AMI for 10 mmHg lower usual SBP in 60-69 years group (0.709) was estimated from RR of all ischemic heart disease (0.719, from Log(RR) 0.33 in Table S1 in Singh GM et al) [7,9,10].

**1-year RRs estimation**

Based on these RRs derived from APCSC study with average 7-year follow-up, the SBP reduction of 5 mmHg in the base case corresponded to an annual RR of AMI 0.9757 and RR of stroke 0.9670 using formula: Exp[Ln(RR_total) ÷ years]. Similarly we derived other RRs in Table 3 in the manuscript for all scenarios.

**Section 5. Transition probabilities between states**

**All-cause mortality and disease-specific mortality**

The age-specific all-cause mortality rate and CVD-specific mortality in the general Chinese population were obtained from national disease surveillance data reported in China Health and Family Planning Yearbook 2015 [2] and weighted by the population in urban and rural (S1 Table 1).

**S1 Table 1.** Per annum age-speciﬁc all-cause mortality in general Chinese population in 2014

| Age, years | All-cause Mortality  (/100,000) | AMI-specific mortality  per annum (/100,000) | Stroke-specific mortality  per annum (/100,000) |
| --- | --- | --- | --- |
| 65-69 | 1705 | 152.5 | 306.2 |
| 70-74 | 2722 | 255.4 | 513.2 |

**Incidence** **of total stroke**

Age-specific incidences of stroke for modelling were listed in S1 Table 2. S1 Table 2 presented how we estimated the age-specific incidence of total stroke. As the national representative data amongst Chinese on incidence of stroke is scarce, we first calculated the incidence by ischemic and hemorrhagic stroke by dividing age-specific mortality by mortality-to-incidence ratio (MIR) in Chinese, then summed it up by age group to that of total stroke. The age-specific mortality of stroke was obtained from the China Health and Family Planning Yearbook 2015 [2]. The MIRs listed in S1 Table 2 were derived from Global Burden of Diseases, Injuries, and Risk Factors Study 2010 (GBD 2010) [11,12] as shown in S1 Table 3.

**S1 Table 2.**

|  |  | Mortality-to-incidence ratio  C1 | Mortality per annum (/100 000)  C2 | **First-ever incidence per annum**  **(/100 000)**  **C3 =C2/C1** | **Inflated to fit the total mortality**  **(/100 000)** |
| --- | --- | --- | --- | --- | --- |
| 65-69 years | **Total stroke** | - | 306.17 | 1251.05 | 1440 |
|  | Ischemic | 0.132 | 110.54 | 837.45 |  |
|  | hemorrhagic | 0.473 | 195.63 | 413.60 |  |
| 70-74 years | **Total stroke** | - | 513.2 | 2238.25 | 2280 |
|  | Ischemic | 0.132 | 211.16 | 1599.68 |  |
|  | hemorrhagic | 0.473 | 302.04 | 638.57 |  |

As listed in S1 Table 3, GBD 2010 first reported an overall age-adjusted MIR and age-specific MIR for first-ever ischemic stroke and hemorrhagic stroke in low-income and middle-income countries in 2010 (Table 3 in Krishnamurthi RV et al) [11], as well as an overall MIR in all ages in Chinese in 2010 (Table 1 and 2 in Krishnamurthi RV et al) [11]. The MIRs in 65-74 years in Chinese in grey cells were estimated from that in low-income countries and matched the overall MIR in China.

**S1 Table 3. The mortality-to-incidence ratios for ischemic and hemorrhagic stroke from GBD 2010 study to estimate that of 65-74 years in Chinese.**

|  |  | Low-income countries | China in 2010 |
| --- | --- | --- | --- |
| All ages |  |  |  |
|  | Ischemic stroke | 0.223 | 0.194 |
|  | Hemorrhagic stroke | 0.595 | 0.50 |
| 65-74 years |  |  |  |
|  | Ischemic stroke | 0.152 | 0.132 |
|  | Hemorrhagic stroke | 0.563 | 0.473 |

**Incidence of AMI**

The presented transition probabilities for AMI were based on data in early years or some regional data in Chinese and some assumptions. The age-specific incidence rate of AMI was based on 10-year incidence rate from the China Hypertension Survey Epidemiology Follow Up Study (CHEFS), and to derive the 1-year transition probabilities for incidence as in Moran A et al [13]. Taking into account the decline trend of mortality over years, we then calibrated to fit with the national age-specific mortality of AMI in China Health Statistics Yearbook 2015 estimate for 2014 [2] and case-fatality assumptions according to the ratio of mortality to incidence from CHEFS [13].

**Case-fatality rate of total stroke**

The 28-day case-fatality rate of total stroke were derived from Beijing Sino-MONICA data [14,15], which indicated the overall estimates in 35–74 years of that in 1984–2000 was 28.76%, with 34.1% in 65–74 years, and appeared an absolute decline of annual 1.63% in overall case-fatality in total stroke from 1984–2000 [14,15]. After accounting for the above annual decline rate, we estimated the case-fatality for total stroke in 65–74 years was about 20% in the end year of the study 2000. In the base case, we then assumed a 20% case-fatality of total stroke.

**Case-fatality rate of AMI**

The 28-day case-fatality rate of AMI was estimated from pooled Beijing Sino-MONICA study data from 1999-2004 from appendix figure 1 in Moran A et al [13] In which, reported the 28-day case-fatality rates for ages 25–74 years from 1999-2004 were 40% for men and 49% for women. The main age-specific AMI case-fatality rate assumptions were estimated from the overall rates [appendix Table 8 for 1993-2004 in Moran A et al] [13] and we proportionally estimated that that in ages 65-74 years it was 44%.

**Transition probabilities assumptions in chronic CVD state**

For people in the chronic CVD state, the death risk from other causes in AMI survivors and stroke survivors was assumed to be 1.2 (range of 1 to 1.5) times of that in the well state, considering the people in chronic CVD state is more likely to die from other causes because of the worse condition than in well state. Additionally, we assumed that the risk for recurrent AMI or stroke of a person in the chronic CVD state, given prior AMI or stroke, was 4 times (range of 3 to 5) of that in the well state. The 28-day case-fatality rate of recurrent events was assumed to be 1.4 times (range of 1 to 2) of that in first-ever events, informed by Beijing-MONICA study (Table 3 in Sun J et al, 2007) [15].

**Section 6. References**

1 Ministry of Education of the People’s Republic of China. Statistical data. Available from: <http://www.moe.edu.cn/>. Accessed October, 2016.

2 National Health and Family Planning Commission of China. China Health and Family Planning Yearbook 2015. Beijing: Peking Union Medical College Press. 2015. (Chinese)

3 Yang L, Wu M, Cui B, Xu J. Economic burden of cardiovascular diseases in China. *Expert Rev* *Pharmacoecon Outcomes Res.* 2008; **8(4):** 349–356.

4 Trading Economics. Available from: <http://www.tradingeconomics.com/china/inflation-cpi>. Accessed December, 2015

5 Smith-Spangler CM, Juusola JL, Enns EA, Owens DK, Garber AM. Population strategies to decrease sodium intake and the burden of cardiovascular disease: a cost-effectiveness analysis. *Ann Intern Med.* 2010; **152:** 481–487, W170–173.

6 Tengs TO, Wallace A. One thousand health-related quality-of-life estimates. *Med Care.* 2000; **38:** 583–637.

7 Asia Pacific Cohort Studies Collaboration. Blood pressure and cardiovascular disease in the Asia Pacific region. *J Hypertens.* 2003; **21:** 707–716.

8 Lewington S, Clarke R, Qizilbash N, Peto R, Collins R. Prospective Studies Collaboration. Age-specific relevance of usual blood pressure to vascular mortality: a meta-analysis of individual data for one million adults in 61 prospective studies. *Lancet.* 2002; **360:** 1903–1913.

9 Lawes CM, Bennett DA, Feigin VL, Rodgers A. Blood pressure and stroke: an overview of published reviews. *Stroke.* 2004; **35:** 1024–1033.

10 Singh GM, Danaei G, Farzadfar F, Stevens GA, Woodward M, Wormser D, et al. The age-specific quantitative effects of metabolic risk factors on cardiovascular diseases and diabetes: a pooled analysis. *PLoS ONE.* 2013; **8(7):** e65174.

11 Krishnamurthi RV, Feigin VL, Forouzanfar MH, Mensah GA, Connor M, Bennett DA, et al. Global and regional burden of first-ever ischaemic and haemorrhagic stroke during 1990-2010: findings from the Global Burden of Disease Study 2010. *Lancet Glob Health.* 2013; **1(5):** e259–281.

12 Feigin VL, Forouzanfar MH, Krishnamurthi R, Mensah GA, Connor M, Bennett DA, et al. Global and regional burden of stroke during 1990-2010: findings from the Global Burden of Disease Study 2010. *Lancet.* 2014; **383:** 245–254.

13 Moran A, Zhao D, Gu D, Coxson P, Chen CS, Cheng J, et al. The Future Impact of Population Growth and Aging on Coronary Heart Disease in China: Projections from the Coronary Heart Disease Policy Model-China. *BMC Public Health.* 2008; **8:** 394.

14 Zhao D, Liu J, Wang W, Zeng Z, Cheng J, Liu J, et al. Epidemiological transition of stroke in China: twenty-one-year observational study from the Sino-MONICA-Beijing Project. *Stroke.* 2008;**39:** 1668–1674.

15 Sun J, Zhao D, Wang W, Liu J, Cheng J, and Jia Y. The changing case-fatality of acute stroke in Beijing during 1984-2000. *Chin J Intern Med.* 2007; **46(5):** 362–365.
